# The prognostic and diagnostic use of microRNA expression in chronic HIV infection

**DOI:** 10.1186/1742-4690-9-S2-P166

**Published:** 2012-09-13

**Authors:** L Zhu, C Qiu, C Ma, X Zhang, J Xu

**Affiliations:** 1 Fudan University, Shanghai Public Health Clinical Center, Shanghai, China; 2 Chinese Academy of Sciences, Shanghai, China; 3 Fudan University, Institutes of Biomedical Sciences, Shanghai, China

## Background

MicroRNA (miRNA) is engaged in the regulation of host immunity during HIV infection and may play an important role in the disease progression. We sought to identify a diagnostic/prognostic miRNA signature to diagnose/predict the outcome of chronic HIV infection.

## Methods

The expression levels of 754 known human microRNA were quantified in whole peripheral blood by TaqMan low density array for 23 chronically HIV-infected subjects. Significance analyses of miRNA expression profiles were performed on groups with varied levels of CD4+ T cell counts or viral loads. The identified prognostic miRNA signature was further validated in a 51 HIV+ patient cohort naïve to ART with following up for 2 years. In addition, we assessed their association with progression-free rate. For the latter, various prediction algorithms were computed on the basis of weighted levels of the miRNAs forming the outcome signature.

## Results

Of the 10 differentially expressed miRNAs (p<0.001), 3 were validated to contribute to the predictive value and thereby used as prognostic signature. Based on different prediction models (binary discrete model and linear models), the prediction accuracy ranged from 84.31% to 88.24% compared with subsequent 2-year follow-up. When applied all the computed classifiers to serial time-point samples of seven progressors and five non-progressors collected during the 2-year follow up, binary discrete model show a better prediction stability. Low miR-31, miR-29b, miR-590-5p were associated with poor progression-free rate, independent of age, gender and known prognostic factors including viral load and immune activation.

## Conclusion

A unique microRNA signature has been associated with advance disease and identified as potential prognostic marker, and provides a new strategy to select patients who would benefit from earlier ART.

